# Morbid obesity in a young woman affected by advanced chronic kidney disease: an exceptional case report. Does a high dose of essential amino acids play a key role in therapeutic success?

**DOI:** 10.1038/nutd.2016.4

**Published:** 2016-02-29

**Authors:** S Caria, S Murtas, G Loria, F S Dioguardi, R Secci, P Bolasco

**Affiliations:** 1 S.C. Territoriale di Nefrologia e Dialisi, ASL Cagliari, Quartu Sant'Elena, Italy; 2 Anestesia e Rianimazione, Ospedale Marino, ASL Cagliari, Quartu Sant'Elena, Italy; 3 Dipartimento di Scienze Cliniche e di Comunità-Università degli Studi Milano, Milano, Italy

## Abstract

A 38-year-old woman, obese (219 kg), diabetic, hypertensive, chronic kidney disease (CKD) stage 4, with low plasma albumin level (2.9 g dl^−1^) and marked proteinuria (22 g per day) was studied. Given the advanced-stage CKD with nephrotic proteinuria, we supplemented low-protein diet with high doses of a tailored essential amino acid mixture (AAs: 44 g per day) to improve weight reduction in the patient. After 20 months of conservative therapy, the patient lost 43 kg; despite two episodes of infection, albumin plasma levels increased up to 3.7 g per day. After a further 20 months of dialysis, the patient maintained a diet of 1800 kcal supplemented with 32 g of AAs and lost 47 kg, whereas both albumin (3.89±0.12 g dl^−1^) and C reactive protein returned to normal. During the follow-up period, anemia improved, erythropoietin was thus discontinued and insulin requirement decreased to 105 IU. This therapeutic option may be beneficial in advanced CKD patients with obesity and diabetes resulting from malnutrition.

## Introduction

Diabetic patients with a body mass index (BMI) >35 kg m^−^^2^ feature a seven-fold higher risk of developing chronic kidney disease (CKD) and hypertensive patients a six-fold higher risk.^1^ The predictive power of abdominal obesity for progression of kidney failure and onset of cardiovascular events is particularly strong;^2, 3^ in patients in dialysis it is also associated with a high risk of death.^4^

We report the case of a young woman affected by severe badly nourished obesity, diabetes, arterial hypertension and advanced CKD. Moreover, we highlight the peculiarity of the measures adopted in clinical, diagnostic and therapeutic management.

## Case report

The patient had been affected by abdominal obesity since childhood, reaching a weight of 110 kg during puberty. At the age of 16 years, she went on a high-protein diet with amphetamine supplements and lost 50 kg. Over the following years, she attempted a series of diets and underwent implantation of a gastric balloon. However to no avail, as she progressively gained weight.

The patient, affected by arterial hypertension, insulin-dependent type 2 diabetes, diabetic retinopathy and Hashimoto's thyroiditis, came to our observation in October 2011, at the age of 38 years, for CKD stage 4 with glomerular filtration rate (GFR) 21 ml min^−^^1^, serum creatinine (sCr) 3.9 mg dl^−1^, blood urea nitrogen (BUN) 53 mg dl^−1^, abdominal obesity (219 kg, waist circumference 190 cm, hip circumference 180 cm), BMI 81 kg m^−^^2^, nephrotic proteinuria (22 g per day), hypoalbuminemia (2.9 g dl^−1^), dyslipidemia and secondary hyperparathyroidism (SHPT) (Table 1). sCr levels were normal until 2007 (0.8 mg dl). To exclude other causes of renal diseases an immunological panel examination was performed, revealing negative findings. Renal biopsy could not be carried out, as the kidneys were embedded in an extensive layer of thick fat. Hypothyroidism was successfully managed with levothyroxine, and cardiac ejection fraction was normal (60%). The patient had poor blood pressure control (200/110 mm Hg), therefore antihypertensive therapy was increased by supplementing the angiotensin-converting-enzyme inhibitor with calcium channel blockers, β-blockers, doxazosin and angiotensin receptor blockers; the latter were also added to enhance the anti-proteinuric effect. Statins could not be administered due to intolerance. Although glycated hemoglobin (HbA1c) level was normal (Table 1), glycemic control was poor, the patient was therefore given high doses of insulin: 130 IU. Given the advanced CKD, a low-protein diet (LPD) was suggested; the patient refused to follow a very low-protein diet, but agreed to an LPD: by food intake recall, it was revealed that the patient was introducing 2700 kcal per day, and consequently caloric intake was reduced by 500 kcal.^5, 6^ The diet consisted of the following: carbohydrates 58%, lipids 32%, proteins 10%, phosphorus 895 mg, sodium 752 mg and potassium 2100 mg. In view of the inadequacy of nutrition, advanced CKD, nephrotic proteinuria and insulin resistance, and with the aim of increasing weight loss, we finally supplemented the diet with high doses of AAs: one sachet of special AAs mixture (4 g: L-Leucine 1250 mg, L-Lysine 650 mg, L-Isoleucine 625 mg, L-Valine 625 mg, L-Threonine 350 mg, L-Cistine 150 mg, L-Histidine 150 mg, L-Phenyl-alanine 100 mg, L-Methionine 50 mg, L-Tyrosine 30 mg and L-Tryptophan 20 mg (Aminotrofic, Errekappa Euroterapici, Milano, Italy) per 20 kg of actual body weight. The singular composition of this AAs mixture was determined in a particular stoichiometric ratio, and contained all essential amino acids and two non-essential amino acids (tyrosine and cysteine).

At this point, although the patient was able to manage her personal needs, she was only able to walk a few meters unaided.

**Figure 1:**
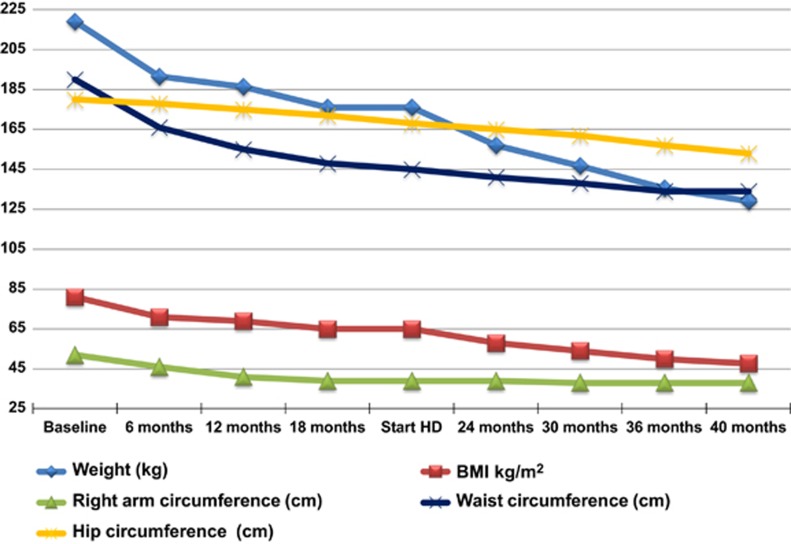
Anthropometric parameters.

Table 1 shows the outcome of laboratory examinations over a period of 40 months. Figure 1 took into account the following anthropometric parameters: body weight, BMI, circumference of the arm, and circumference of the hip and waist.^7^ The patient began taking a dietary supplement of 44 g AAs divided into four daily doses in November 2011 (baseline). Within 3 months, the patient lost 10 kg, although her kidney function was stable (sCr 3.6 mg dl^−1^, BUN 40 mg dl^−1^ and GFR 24 ml min^−1^). Following these results, the patient was strongly motivated to continue losing weight and in February 2012 (4th month) because of progressive respiratory failure with hypoxia and need to evaluate cardiovascular risk, the patient was admitted to hospital and started on low-calorie enteral nutrition (EN) via a nasal feeding tube to increase the speed of weight loss: 2000 ml of liquid per day, 56 g of AAs per day, electrolyte and vitamin supplements, and no food intake. Within 10 days, she lost 21 kg while her kidney function remained stable: sCr 3.3 mg dl^−1^, BUN 36 mg dl^−1^, GFR 24 ml min^−1^, proteinuria/24 h 16 g, plasmatic bicarbonate level 22 mEq l^−1^ and albumin level 3.0 g dl^−1^. Because of a condition of respiratory insufficiency, continuous O_2_ therapy was also prescribed. When EN was stopped, the patient restarted a supplemented LPD. In the 5th and the 15th months, the patient presented with a complication in the form of an infected ulcer in the first toe of her left foot, which was treated with protracted antibiotic therapy, but was resulted in persistently high CPR levels. In the 15th month O_2_ therapy was discontinued due to the resolution of respiratory insufficiency. After 20 months of conservative therapy, the patient had lost a total of 43 kg (Figure 1), and despite the persistent increase in C reactive protein (CRP), albumin levels gradually improved to 3.7 g dl^−1^ (Table 1). In the 20th month, due to worsening of kidney functions, the patient started hemodialysis (HD), particularly to correct peripheral edemas and poorly controlled treatment-resistant arterial hypertension. During hemodialysis the patient's diet was changed and protein-free products removed, although it continued to be supplemented with AAs (32 g per day, reduced depending on the weight reached: 176 kg): 1800 kcal, carbohydrates 53%, lipids 30%, proteins 17%, phosphorus 900 mg, potassium 2200 mg and sodium 1462 mg. After the first 3 months, the patient received a 3 weekly bicarbonate diffusive HD without acetate for 15–16 h per week. Satisfactory dialysis efficiency (eKT/V 0.8±0.1) could not be reached due to the high body surface of the patient, nevertheless a good metabolic balance and nutritional status were achieved (Table 1); after 20 months of dialysis, the patient had lost a further 47 kg and reached a weight of 129 kg. The patient referred to being anuric accordingly, equilibrated protein catabolic rate was calculated based on the actual weight of the patient, and was found to be stable: 1.0±0.2 g kg^−1^ per day.^8^ Throughout the observation period, bicarbonate level was normal.

## Discussion

The patient lost a total of 90 kg over a 40-month follow-up period, with a ΔBMI of 33.3 kg m^−^^2^, her waist circumference decreased by 56 cm, arm circumference by 14 cm and hip circumference 27 cm; albumin levels increased from 2.9 to 3.8 g dl^−1^, her insulin requirement decreased by 105 IU and the patient maintained a good metabolic balance over time (Table 1; Figure 1). The patient's diet was reduced from 2200 kcal of the conservative phase to 1800 kcal in HD; in our opinion, this kcal reduction alone was not sufficient to justify the weight loss registered, particularly as when the patient weighed 219 kg she was not successful in adhering to a series of low-calorie diets, and was moreover unable to undertake physical exercise. As previously reported by Teplan V *et al.*,^9^ the major contribution to the patient's weight loss was provided by AAs, which the patient received in high doses. These compounds ensure a correct protein intake, increase only lean body mass, reduce insulin resistance by increasing activity of the cellular glucose transporter GLUT4 (which reduces blood glucose levels by acting in an insulin-independent manner), and increase the utilization of lipid and glucose substrates.^10, 11, 12, 13, 14, 15^ AAs moreover increase the production of adiponectin, leading in turn to a rise in insulin sensitivity.^16, 17^ According to the literature, the use of AAs in our patient caused a sharp reduction in the need for exogenous insulin, whereas increasing the efficiency of endogenous insulin, as had already been observed in patients in highly critical conditions.^18^ In the particular AAs mixture formulation administered, the branched chain amino acids (BCAA) make up about 50% of total AAs. The resulting BCAA blood increase activates beta-oxidation, which enables the utilization of lipids for energy.^19^ Indeed, AAs regulate carbohydrate and fat metabolisms, thus contributing towards maintaining the basal metabolism by reducing fatty acid synthesis in the cytoplasm and providing for an augmented access into the mitochondria.^20^ Leucine, which the patient received at high doses (13.75±g per day), is essential for the promotion of protein anabolism and inhibition of their catabolism,^21, 22, 23^ and reduces appetite at the level of the hypothalamus.^24^ Our patient indeed confirmed that she had experienced a reduction in the sensation of hunger. In our opinion, the above-mentioned characteristics of AAs account for the remarkable weight reduction observed. In the patient, high doses of AAs met nitrogen requirements by minimizing urea synthesis due to the absence of arginine into the amino acids formulation, provided for an adequate caloric intake and produced a good nitrogen balance throughout the observation period, without eliciting protein catabolism. During the follow-up period, BUN remained within an acceptable range (Table 1); this depended both on the fact that a significant part of the produced azotemia was due to the inclusion of arginine in the patient's diet, and the improved action of non-essential amino acids on protein biosynthesis which, due to the high levels of AAs, was not catabolized into urea.^25^

Nevertheless, the progression of CKD was unfavorable; even though a good metabolic balance was achieved, residual kidney function gradually but progressively decreased (−0.4 ml min^−1^ per month). This may have been due to a series of factors such as severe arterial hypertension, diabetes, obesity, persistent inflammatory status, high proteinuria and/or a potentially undiagnosed additional renal disease.^26^

Dyslipidemia improved with a progressive reduction of low-density lipoprotein (Table 1), in spite of the patient not receiving statins; this effect may likewise have been the result of both diet and AAs supplementation.^27^ Serum phosphate levels were well controlled throughout the observation period, due to the lack of phosphate in the AAs mixture. Severe SHPT persisted and was treated with paricalcitol 2.0 mcg per day during conservative therapy and 2.6±0.6 mcg per day during dialysis; after 40 months chronic renal disease-mineral bone disorders therapy had produced a decrease in intact parathyroid hormone to 102 pg ml^−1^ (Table 1). Anemia improved progressively, and ultimately the administration of erythropoietin was discontinued (Table 1). During the 20 months of dialysis, the patient did not contract any infections, CRP remained at lower levels compared with the conservative phase (CRP 10.4±1.7 versus 17.4±3.9 mg l^−1^), whereas albumin levels stabilized (3.89±0.12 g d l^−1^). Administration of antihypertensive drugs was discontinued a few months after commencing HD in view of improved interdialytic weight loss (average 3.0% of body weight), progressively extended duration of dialysis session, and better modulation of dialysate sodium, assessed on the basis of blood pressure levels ranging between 140–142 mmol l^−1^. During the conservative phase, the patient received 300 mg die^−1^ of allopurinol, although she failed to achieve target values of uric acid (Table 1). In spite of the difficulties encountered in achieving a good dialysis efficiency, high-protein intake (respect to the conservative phase) and discontinuation of allopurinol at start of dialysis, uric acid levels decreased gradually, reaching values of 7.1 mg d l^−1^ in the 40th month (Table 1). It might be expected, therefore a possible role of the AAs, probably when taken continuously over a long period of time in the inhibition of the synthesis of uric acid.

At the end of the follow-up period, the patient's quality of life had improved remarkably, and she was able to walk independently again. The patient had showed a good compliance in following the diet and taking AAs. The patient has recently been moved to the plastic surgery unit to undergo remodeling of the abdomen with removal of all fat deposits, with an estimated weight loss of an additional 30 kg approximately. The goal is to achieve an overall weight loss of 130 kg (from 219 kg to 89 kg).

## Figures and Tables

**Table 1:** Outcome of nutritional and functional parameters

	*Baseline*	*6th month*	*12th month*	*18th month*	*Start HD* *20th month*	*24th month HD*	*30th month HD*	*36th month HD*	*40th month HD*
Crs (mg dl^−1^)	3.9	3.2	3.9	5.7	6	6.5	6.9	8	9.7
BUN (mg dl^−1^)	53	61	66	74	88	85	82	80	80
GFR (ml min^−1^)^a^	21	18.5	15	13.5	13	—	—	—	—
eGFR (ml min^−1^)^b^	13.8	17.5	13.7	8.65	8.13	—	—	—	—
eGFR (ml min^−1^)^c^	13.7	17.2	13.7	8.8	8.3	—	—	—	—
Proteinuria (g per day)	22	13	12	10	8	—	—	—	—
Potassium (mmol l^−1^)	6.1	5.5	4.8	5	4.6	4.4	4.2	4.8	5.3
Calcium (mg dl^−1^)	8.8	9.6	9.7	8.84	9.2	8.86	8.9	8.7	8.86
Phosphorus (mg dl^−1^)	4.7	3.8	4.2	4.4	5.5	5.3	5.4	5	3.9
PTH (ρg ml^−1^)	707	245	484	559	508	445	579	600	102
CRP (mg l^−1^)^d^	16	16	23	11	11	10	12	7	10
Total proteins (g dl^−1^)	6.9	7	7.4	7.7	7.8	7.6	7.7	7.5	7.3
Albumin (g dl^−1^)	2.9	3.35	3.3	3.7	3.7	3.92	4	3.9	3.8
Total cholesterol (mg dl^−1^)	285	190	177	164	151	145	140	146	127
Triglycerides (mg dl^−1^)	216	168	102	129	107	130	176	155	151
LDL cholesterol (mg dl^−1^)	199	122	121	101	99	88	77	87	71
HDL cholesterol (mg dl^−1^)	43	34	36	37	31	31	28	28	28
Lynphocytes per mm^3^	1584	1711	1480	1500	1500	1550	1712	1891	1920
Transferrine (mg dl^−1^)	281	216	240	261	225	245	262	247	254
Uric Acid (mg dl^−1^)	9.8	6.5	5.8	7.2	8	8.5	8.3	8.1	7.1
HbA1c (%)	6.5	6.3	6.2	6.4	6.3	6	6.4	6.4	6.5
Hb (g dl^−1^)	12	9.3	10.6	11.2	11	10.4	11.8	13.8	14
EPO (U kg^−1^ per week)	—	104	107	110	35	39	13.7	—	—
ERI (U kg^−1^ per week)/Hb	—	11	10	9.8	3.2	3.7	1.2	—	—

Abbreviations: BUN, blood urea nitrogen; CKD, chronic kidney disease; Crs, serum creatinine; eGFR, estimated glomerular filtration rate; EPO, erythropoietin; ERI, erythropoietin resistance index; GFR, glomerular filtration rate; Hb, hemoglobin; HbA1c, glycated hemoglobin; HD, hemodialysis; HDL, high-density lipoprotein; LDL, low-density lipoprotein; PTH, parathyroid hormone.

a GFR: measured as the average of creatinine clearance and urea clearance, and expressed as ml/min*1.73 m2 body surface area.

b Estimated by CKD-EPI formula.

c Estimated by Modification of Diet in Renal Disease (MDRD) formula.

d CRP normal value ⩽10.
